# Assessing the effectiveness and safety of Patisiran and Vutrisiran in ATTRv amyloidosis with polyneuropathy: a systematic review

**DOI:** 10.3389/fneur.2024.1465747

**Published:** 2024-09-02

**Authors:** Mohammad Amin Karimi, Fatemeh Esmaeilpour Moallem, Mohammad Sadra Gholami Chahkand, Eftekhar Azarm, Mohammad Javad Emami Kazemabad, Parisa Alsadat Dadkhah

**Affiliations:** ^1^School of Medicine, Shahid Beheshti University of Medical Sciences, Tehran, Iran; ^2^Student Research Committee, Golestan University of Medical Sciences, Gorgan, Iran; ^3^School of Medicine, Isfahan University of Medical Sciences, Isfahan, Iran; ^4^Student Research Committee, School of Medicine, Qom University of Medical Sciences, Qom, Iran

**Keywords:** hereditary amyloidosis, transthyretin-related amyloidosis, Patisiran, Vutrisiran, polyneuropathy, cardiomyopathy, clinical trials

## Abstract

**Background:**

Hereditary transthyretin (ATTRv) amyloidosis, a multifaceted disorder affecting multiple systems, substantially diminishes patients’ physical capabilities and overall quality of life. Patisiran and Vutrisiran, two Ribonucleic acid (RNA) interference therapies, target reducing both pathogenic and wild-type transthyretin (TTR) protein levels. This systematic review assesses the effectiveness and safety of these treatments in managing ATTRv.

**Methods:**

A comprehensive, thorough literature search across databases including Embase, PubMed, Web of Science, Cochrane Central, and Google Scholar yielded 858 studies. Following removing duplicate and irrelevant articles, 676 distinct studies underwent review. These studies, conducted on a global scale, encompassed a range of methodologies, including clinical trials and indirect treatment comparisons.

**Results:**

Ten studies, spanning a total population of 756 patients, were selected for in-depth analysis. Patisiran and Vutrisiran consistently demonstrated significant improvements in primary and secondary endpoints related to neuropathy, quality of life, and cardiac function. Both medications were well-tolerated, with primarily mild to moderate adverse events. Indirect treatment comparison studies indicated Vutrisiran’s superiority over Tafamidis in treating ATTRv amyloidosis.

**Conclusion:**

This systematic review recommends using Patisiran and Vutrisiran to treat ATTRv amyloidosis. The findings suggest that these RNA interference therapies improve neuropathy, quality of life, and cardiac symptoms. The results indicate sustained benefits over prolonged treatment, with satisfactory safety profiles. However, potential biases, conflicts of interest in the studies, and limited follow-up periods in some trials necessitate cautious interpretation. Future research should address these limitations and provide more robust evidence for the long-term efficacy and safety of Patisiran and Vutrisiran in ATTRv treatment.

## Introduction

Hereditary transthyretin (ATTRv) amyloidosis is a disease typically manifesting in adulthood, subsequent to mutations within the transthyretin (TTR) gene. TTR protein constitutes a circulating tetramer serving as a carrier protein for thyroxine and retinol-binding proteins affiliated with vitamin A. TTR is detectable in both serum and cerebrospinal fluid, predominantly biosynthesized in hepatic tissue, with minor synthesis occurring in the eye and the brain (1). Genetic mutations within the TTR gene precipitate the destabilization of its tetrameric structure, resulting in the dissociation into misfolded monomers, which aggregate within extracellular spaces, forming oligomers that eventually coalesce into amyloid fibrils typified by cross β-sheets (2). Accumulation of mutated transthyretin primarily occurs in the heart and peripheral nervous system (PNS), with secondary involvement of various organs such as the brain, kidneys, skin, and muscles, rendering ATTRv a multisystemic disorder. Over 130 pathogenic mutations have been delineated, with the most common TTR variants being the valine-to-methionine substitution at amino acid 30 mutation (Val30Met) and the Val122Ile mutation. However, early-onset disease is not the typical phenotype in all endemic areas. Late-onset disease is more common in Sweden, and this phenotype is also present in Japan and Portugal (3). Other mutations, particularly non-Val30Met variants, predominantly present with delayed manifestation characteristics (4). Primary clinical manifestations of ATTRv predominantly affecting the PNS or cardiac system delineate distinct neurological or cardiac phenotypes. However, most mutations progressively manifest as a mixed phenotype involving both systems throughout the disease course (1).

ATTRv polyneuropathy emerges due to the accumulation of TTR within the PNS, leading to the development of sensory-motor and autonomic neuropathies characterized by length-dependent patterns. Manifesting as a progressive and incapacitating condition, untreated ATTRv results in a worsening disability, ultimately culminating in wheelchair dependence, bedridden states, and eventual mortality (5). Therefore, early identification of individuals affected by ATTRv is imperative, and prompt initiation of treatment is vital to enhance their quality of life (QOL) and attain favorable clinical outcomes (6).

Therapeutic modalities for ATTRv amyloidosis encompass a range of interventions, including transthyretin tetramer stabilizers such as Tafamidis, pharmacotherapies targeting transthyretin reduction like Patisiran, and surgical interventions such as liver transplantation (7, 8). Patisiran and Vutrisiran represent two newly approved pharmaceutical agents designed to mitigate abnormal protein levels through mechanisms involving either stabilization of protein structure or inhibition of protein production. These medications are classified as RNA interference (RNAi) therapies, explicitly targeting the TTR gene and effectively diminishing its expression within the liver (9–12). Both pharmaceutical compounds have demonstrated effectiveness and safety through clinical trials involving individuals afflicted with ATTRv accompanied by polyneuropathy. These medications have obtained regulatory approval from both the US Food and Drug Administration (FDA) and the European Medicines Agency (EMA) (13–15). However, these drugs have differences and discrepancies regarding their mode of action, pharmacokinetics, and clinical outcomes.

Medications like Inotersen and Eplontersen are anti-sense oligonucleotides and mediate RNA cleavage (16, 17). They target the mRNA of the transthyretin gene and reduce the production of abnormal proteins that are responsible for ATTRv amyloidosis (17, 18). Activation of RNase H by Inotersen causes cleavage of the mRNA that is responsible for transthyretin coding (18). Eplontersen attaches to both normal and mutant TTR mRNA and causes their degradation. This process inhibits TTR protein production, preventing amyloid deposits (17). Both Inotersen and Eplontersen have been recently approved for the treatment of polyneuropathy in patients with hereditary transthyretin-mediated amyloidosis (ATTRv amyloidosis) (17, 18).

This systematic review aims to critically examine and compare the cardiologic, neurologic, and adverse events of Patisiran and Vutrisiran on ATTRv with polyneuropathy.

## Methods

### Study protocol

Our systematic review assessed the efficacy of novel drugs for polyneuropathy in ATTRv amyloidosis (Patisiran and Vutrisiran). The current investigation followed the guidelines specified within the Preferred Reporting Items for Systematic Reviews and Meta-Analysis (PRISMA) framework to conduct a thorough systematic review and meta-analysis in accordance with established protocols (19). The design protocol for our study was appropriately registered in the Open Science Framework (OSF). The hyperlink supplied leads to the OSF database, which houses a unique identification number record.^1^

### Literature search

In the initial stage, relevant keywords were determined using Medical Subject Headings (MeSH) terminology. Subsequently, a thorough inquiry was conducted till September 16, 2023. The investigation utilized the following databases: PubMed, Google Scholar, Scopus, Web of Science, and Cochrane Central. The search strategy was executed by considering the title, abstract, and pertinent keywords and tags, utilizing sophisticated search functionalities for each search engine. To enhance the accuracy of the search, the search strategy involved three primary subgroups of keywords and MeSH. One subgroup encompassed terms associated with the generic, brand, and premarketing titles of Patisiran and Vutrisiran. The second subgroup included keywords and terminology of Amyloid Neuropathies. Finally, the subgroups were merged using the ‘AND’ operator. No specific limitations or restrictions were placed on the date, type of publishing, or language. The search methodology utilized in this investigation is illustrated in Supplementary Table 1.

### Study selection

This research is dedicated to examining the criteria for inclusion and exclusion within the research framework. The investigators utilized specific parameters to identify suitable publications. The eligibility requirements for this study were delineated as follows:

- 1. The study design needed to encompass observational cohort studies (both retrospective and prospective), randomized controlled trials, or early-phase clinical trials. These studies must be published in peer-reviewed journals and comprehensively analyzed.
- 2. The trials under investigation must have evaluated Patisiran and Vutrisiran as interventions for Amyloidosis.
- 3. The current study incorporated research inquiries that delved into the comparative efficacy of Patisiran or Vutrisiran compared to placebo, active therapies, or no intervention among adults aged 18 years or older.
- 4. Our study comprised investigations documenting at least one occurrence of neurological or cardiologic outcomes or adverse events.

Conversely, the exclusion criteria were defined as follows:

- 1. The surveyed population was confined to patients with ATTRv, excluding those with other types of Amyloidosis.
- 2. Studies evaluating outcomes deemed irrelevant were excluded from the analysis.
- 3. Only well-designed observational cohort studies (including retrospective and prospective), randomized controlled trials, or early-phase clinical trials were considered for inclusion. Studies employing alternative methods, conducted on animal models, displaying biases, or utilizing different study designs were excluded from this analysis.

### Data extraction

The present analysis focused exclusively on observational cohort studies, including retrospective and prospective designs and randomized controlled trials. Early-phase clinical trials published in peer-reviewed journals were also considered, and comprehensive evaluations were conducted. Our main aim was to thoroughly examine scholarly literature that explores the effects of Patisiran and Vutrisiran on the polyneuropathy associated with ATTRv. After an exhaustive search, eliminating duplicate entries, and retrieving relevant articles, two researchers meticulously reviewed the titles and abstracts of selected studies. In instances of inconsistencies, a third author was consulted to facilitate resolution through deliberation. Subsequently, the next phase involved thoroughly analyzing the full text of identified articles to ascertain their precise alignment with the established inclusion criteria. All studies meeting the predetermined inclusion criteria were incorporated into the analysis, and a third reviewer resolved any discrepancies. The two reviewers systematically gathered pertinent study data using a pre-established data collection table. This encompassed details such as the author’s identification, study methodology, year of publication, sample size, duration, and characteristics of the patient groups. Additionally, the researchers collected various patient characteristics, including gender, age, dosage, follow-up duration, ethnicity, and outcomes. The screening procedure is illustrated in the 2020 PRISMA checklist, as shown in Supplementary Figure 1.

### Quality assessment

Three reviewers individually evaluated the full texts of the articles for suitability, employing critical appraisal instruments sourced from the website specified.^2^ They resolved any disagreements by discussion and consultation with the corresponding author. Three reviewers independently collected the data using a predetermined template. This template encompassed the subsequent information: study title, publication year, publication journal, study characteristics (study design, participants, mean age, administered dose, follow-up duration), and primary outcomes (neurologic and cardiologic parameters and adverse events).

## Results

### Study selection and characteristics

A comprehensive search of primary literature databases encompassing Embase, PubMed, Web of Science, Cochrane Central, and Google Scholar identified 858 studies relevant to the present analysis. After removing duplicate items, the dataset was narrowed down to 676 distinct research studies. Subsequently, a meticulous review of titles and abstracts excluded 577 articles considered irrelevant to the research. Consequent analysis was applied to 99 remaining records, involving a comprehensive examination of their complete texts. Out of this group, 89 papers were excluded because they contained irrelevant or unnecessary information, resulting in a final collection of relevant studies for thorough examination. Finally, 10 articles with a total population of 756 were reviewed.

Coelho et al. (20) employed Phase 1 randomized, single-blind, placebo-controlled, dose-ranging trials to assess the safety and dosage response of two RNAi therapies, ALN-TTR01 and ALN-TTR02 (Patisiran). Several research centers across Portugal, Sweden, France, and the United Kingdom actively participated in this study. The recruitment process entailed including 32 patients for ALN-TTR01 and 17 patients for ALN-TTR02, with 56% being male in the former group and all participants being male in the latter group. The mean age recorded for participants in the ALN-TTR01 trial was 32 years, whereas for those in the ALN-TTR02 trial, it was 27 years. The dosage range for ALN-TTR01 was 0.01 to 1.0 mg/kg, and for ALN-TTR02, it was 0.01 to 0.5 mg/kg (20).

The Phase 2 study by Coelho et al. (21) was conducted internationally across seven countries, including the United States, Brazil, France, Germany, Portugal, Spain, and Sweden. The research comprised a cohort of 27 individuals diagnosed with ATTRv, manifesting mild-to-moderate neuropathic symptoms. The median age of the participants was 64, with 18 being male. The participants received intravenous administration of Patisiran at a dosage of 0.3 mg/kg every 3 weeks (Q3W) for a duration of approximately 2 years (21).

The study conducted by Suhr et al. (22) was characterized by its multicenter and international nature, employing an open-label design focusing on multiple-dose escalation in a phase two clinical trial. A total of 29 individuals met the eligibility criteria for participation in the study. The cohort included patients from diverse nationalities, including Brazilian, French, German, Portuguese, Spanish, Swedish, and American. Notably, all enrolled patients identified as of Caucasian ethnicity, with 69% being male, and the average age of the participants was 55.6 years. In the context of this phase 2 investigation, individuals diagnosed with familial amyloid polyneuropathy (FAP) were subjected to two intravenous infusions of Patisiran, each administered at varying doses: 0.01 (*n* = 4), 0.05 (*n* = 3), 0.15 (*n* = 3), or 0.3 (*n* = 7) mg/kg every 4 weeks (Q4W). Additionally, a subset of patients received a dosage of 0.3 mg/kg Q3W, amounting to a cohort of 12 participants (22).

The HELIOS-A was a worldwide, open-label phase 3 clinical trial conducted by Adams et al. (13). In this study cohort, 122 individuals were designated for the Vutrisiran cohort, 42 for the Patisiran reference cohort, and 77 subjects for the external placebo cohort. Notably, there was a significant male predominance observed within the HELIOS-A cohort, comprising 64.6% (*n* = 106), and within the APPOLO cohort (placebo), constituting 75.3% (*n* = 58). The study encompassed participants aged 18 to 85, recruited from 57 different sites across 22 countries. They were randomly assigned to receive either Vutrisiran at a dosage of 25 mg subcutaneously every 3 months (Q3M) or Partisan at a dosage of 0.3 mg/kg intravenously Q3W over a period of 18 months (13).

Merkel et al. (23) carried out a comparative study, utilizing data from phase three randomized controlled trials involving three distinct groups: the Fx-005, HELIOS-A trial, and APOLLO trial cohorts, comprising 125, 164, and 77 patients, respectively. The Fx-005 trial focused on transthyretin amyloidosis patients with polyneuropathy, and the Val30Met in the TTR variant was a multinational, multicenter, randomized, double-blinded, and placebo-controlled study. It enrolled 125 patients in an intention-to-treat (ITT) group, allocated in a 1:1 ratio to receive either 20 mg of Tafamidis orally (*n* = 64) or placebo (*n* = 61) daily for 18 months. The HELIOS-A trial, also a phase three study, was a randomized, worldwide, open-label investigation assessing the efficacy of Vutrisiran in patients with transthyretin amyloidosis and polyneuropathy. This trial involved 164 patients randomly assigned in a 3:1 ratio to receive either Vutrisiran (*n* = 122; 25 mg subcutaneously, Q3M) or Partisan (*n* = 42; 0.3 mg/kg intravenously, Q3W), which served as the reference comparator. The treatment duration was 18 months, with data from the APOLLO trial, a phase 3 study of Patisiran, used as an external placebo in this investigation. The primary objectives of the study were to evaluate the improvement or stability of the Neuropathy Impairment Score in the Lower Limbs (NIS-LL) and changes in the Norfolk Quality of Life-Diabetic Neuropathy (QOL-DN) total score following 18 months of Fx-005 treatment. The HELIOS-A trial assessed changes in the modified Neuropathy Impairment Score + 7 (mNIS +7) from the baseline measurement at either 9 or 18 months, depending on jurisdictional requirements (23).

The original study by Adams et al. (24) was a phase 3 study conducted across multiple international centers, employing randomization, a placebo control, and a double-blind methodology. It enrolled 225 participants from 44 sites situated in 19 different countries. The study cohort comprised 167 male participants, representing 74% of the total sample, with a median age of 62. These individuals were subjected to random assignment, wherein they were allocated to receive either Patisiran or a placebo as part of a meticulously controlled clinical trial. In this study, 148 individuals were administered Patisiran intravenously at 0.3 mg/kg, with each infusion lasting about 80 min. This treatment regimen was administered Q3W for a total duration of 18 months. In contrast, the remaining 77 participants were subjected to a placebo intervention (24).

In a Phase 3b study conducted by Schmidt et al. (25), 24 individuals with ATTRv amyloidosis and post-LT polyneuropathy were investigated. They were administered a 0.3 mg/kg Patisiran intravenous infusion once every 3 weeks for 12 months. The multinational study encompassed participants from seven countries: France, Germany, Italy, Portugal, Spain, Sweden, and the United Kingdom. The participants had an average age of 58.1 and a mean BMI of 23.5. Males accounted for 56.5% (*n* = 13) of the sample, with 95.7% identifying as white. Notably, the majority of participants identified as non-Hispanic or Latino (25).

The original article by Bleecker et al. (26) is a retrospective study conducted across six Belgian neuromuscular reference centers. The study encompassed a cohort of nine patients, with a median age of 63, who underwent treatment with Patisiran. The Partisan administration was conducted via an 80-min intravenous infusion at 300 μg/kg dosage, administered in Q3W intervals as part of an Expanded Access Program (EAP) (26).

The original study by Fontana et al. (27) encompassed 32 participants diagnosed with transthyretin cardiomyopathy (ATTR-CM). The cohort was divided into two distinct groups: one designated as the treatment group consisting of 16 patients, and another identified as the control group with equal participants. The median age of subjects in the treatment group was 62 years, with 13 male participants, while the control group exhibited a median age of 69 years, encompassing 14 male participants. All patients were administered 0.3 mg/kg Patisiran via infusion Q3W. Of the 16 patients, 12 were given Diflunisal, a TTR-stabilizing drug, orally at 250 mg twice daily. The study participants underwent serial monitoring at 12 months through cardiac magnetic resonance, cardiac biomarkers, bone scintigraphy, 6-min walk tests (6MWTs), and echocardiography (27).

Adams et al. (28) conducted an international, open-label extension (OLE) study across 43 medical facilities spanning 19 countries. This investigation enrolled individuals who had successfully concluded either the phase 3 APOLLO trial or the phase 2 OLE parent trials and could tolerate the experimental medication. The eligible participants from both the APOLLO trial (including both the patisiran and placebo cohorts) and the phase 2 OLE trial (Patisiran cohort) were included in this global extension study, receiving intravenous Patisiran at a dosage of 0.3 mg/kg Q3W, with a planned study period of up to 5 years. Recruitment took place between July 13, 2015, and August 21, 2017, with 211 out of 212 eligible patients enrolled, comprising 137 from the APOLLO-Partisan group, 49 from the APOLLO-placebo group, and 25 from the phase 2 OLE partisan group. As of the data cutoff on September 24, 2018, completion rates for the 12-month assessments were 92% (126 out of 137) for the APOLLO-Partisan group, 78% (38 out of 49) for the APOLLO-placebo group, and 100% (25 out of 25) for the phase 2 OLE Patisiran group (28).

The summary of the study’s characteristics is presented in Table 1.

**Table 1:** Characteristics of studies.

Literature title	First author	Publication year	Publication journal	Type of study	Study location	Patients characteristics	Intervention	Sample size	Sex (Female)	Mean age (years)	Follow up duration
Efficacy and safety of vutrisiran for patients with hereditary transthyretin-mediated amyloidosis with polyneuropathy: a randomized clinical trial (13)	D. Adams	2022	Amyloid	phase 3, global, randomized, open-label study	Conducted at 57 sites in 22 countries	Patients with a diagnosis of ATTRv amyloidosis with a documented TTR variant and neuropathy (baseline NIS of 5–130), a PND score of IIIb, a Karnofsky Performance Status score of ≥ 60%, and adequate liver and renal function.	Vutrisiran 25 mg SC Q3M Patisiran 0.3 mg/kg IV Q3W	APOLLO (placebo): 77 HELIOS-A: Vutrisiran (*n* = 122) Patisiran (*n* = 42)	APOLLO (placebo): 19 (24.7%) HELIOS-A: Vutrisiran: 43 (35.2%) Patisiran:15 (35.7%)	APOLLO (placebo): 63 HELIOS-A: Vutrisiran: 60 Patisiran: 60	18 months
Safety and Efficacy of RNAi Therapy for Transthyretin Amyloidosis (20)	T. Coelho	2013	The New England journal of medicine	Retrospective Study	Portugal, France, UK, Sweden	For the ALN-TTR01 phase 1 trial Patients who had a biopsy-proven diagnosis of transthyretin amyloidosis with mild-to-moderate neuropathy	ALN-TTR01 (at doses of 0.01 to 1.0 mg/kg in 32 patients with transthyretin amyloidosis) ALN-TTR02(Patisiran) (at doses of 0.01 to 0.5 mg/kg in 17 healthy volunteers)	Total: 49 ALN-TTR01Trial: 32 ALN-TTR02 trial: 17	ALN-TTR01Trial: 14 (44%) ALN-TTR02 trial: 0	ALN-TTR01Trial: 32 ALN-TTR02 trial: 27	4 weeks
A phase II, open-label, extension study of long-term patisiran treatment in patients with hereditary transthyretin-mediated (hATTR) amyloidosis (21)	T. Coelho	2020	Orphanet Journal of Rare Diseases	Phase II OLE study	USA, Brazil, France, Germany, Portugal, Spain, and Sweden	Patients had biopsy-proven hATTR amyloidosis with evidence of mild-to-moderate neuropathy, and had previously received and tolerated patisiran in the Phase II study (NCT01617967)	Patisiran 0.3 mg/kg IV (over 70 min) Q3W for approximately 2 years	27	9 (33%)	64	Approximately 2 years
Efficacy and safety of patisiran for familial amyloidotic polyneuropathy: a phase II multi-dose study (22)	O. Suhr	2015	Orphanet Journal of Rare Diseases	Open-label, multipledose escalation phase II trial	Brazil, France, Germany, Portugal, Spain, Sweden, and the USA	Patients who had a biopsy-proven diagnosis of transthyretin amyloidosis with mild-to-moderate neuropathy	Patisiran IV was administered at doses of: 0.01 mg/kg (*n* = 4) Q4W 0.05 mg/kg (*n* = 3) Q4W 0.15 mg/kg (*n* = 3) Q4W 0.3 mg/kg (*n* = 7) Q4W 0.3 mg/kg (*n* = 12) Q3W	29	9 (31%)	55.6	Approximately 30 weeks
Indirect treatment comparison (ITC) of the efficacy of vutrisiran and tafamidis for hereditary transthyretin-mediated amyloidosis with polyneuropathy (23)	M. Merkel	2023	Expert Opinion on Pharmacotherapy	Randomized controlled trial	Not explicitly stated, international and multicenter	Patients with ATTRv amyloidosis with polyneuropathy	Vutrisiran 25 mg SC Q3M Tafamidis: 20 mg orally daily Patisiran 0.3 mg/kg IV Q3W	FX-005 trial: 125 HELIOS-A trial:122 APOLLO trial: 77	Not mentioned	Not mentioned	18 months
Patisiran, an RNAi Therapeutic for Hereditary Transthyretin Amyloidosis (24)	D. Adams	2018	The New England Journal of Medicine	Randomized, double-blind, placebo-controlled, phase 3 trial	Carried out at 44 sites across 19 countries	Patients with hATTR amyloidosis with polyneuropathy	Patisiran 0.3 mg/kg IV Q3W for 18 months Placebo Q3W for 18 months	Total:225 Patisiran:148 Placebo: 77	Total: 58 (25.8%) Patisiran:39 (26%) Placebo:19 (25%)	Total: 62 Patisiran: 62 Placebo: 63	18 months
Patisiran treatment in patients with hereditary transthyretin mediated amyloidosis with polyneuropathy after liver transplantation (25)	H. Schmidt	2022	American Journal of Transplantation	Phase 3b, open label trial	10 centers across France, Germany, Italy, Portugal, Spain, Sweden, and the UK	Patients with ATTRv with polyneuropathy progression post-liver transplantation	Patisiran 0.3 mg/kg IV Q3W for 12 months	23	10 (43.5%)	58.0	12 months
A retrospective survey of patients with hereditary transthyretin-mediated (hATTR) amyloidosis treated with patisiran in real-world clinical practice in Belgium (26)	JL. De Bleecker	2023	Acta Neurologica Belgica	Retrospective Study	Belgium	Patients with hATTR amyloidosis confirmed by genetic testing, with polyneuropathy stage 1 or stage 2	Patisiran 0.3 mg/kg IV Q3W	12	Not mentioned	63	Median Follow up duration: 663 (564–790) days
Reduction in CMR Derived Extracellular Volume With Patisiran Indicates Cardiac Amyloid Regression (27)	*M. Fontana*	2021	Journal of the American College of Cardiology	Prospective observational study	UK	Patients with hereditary ATTR-CM	Patisiran 0.3 mg/kg IV Q3W	Total:32 Patisiran:16 Control: 16	Patisiran:3 (18.7%) Control: 2 (12.5)	Patisiran: 62 Control: 69	12.4 months
Long-term safety and efficacy of patisiran for hereditary transthyretin-mediated amyloidosis with polyneuropathy: 12-month results of an open-label extension study (28)	D. Adams	2021	The Lancet. Neurology	Global OLE trail	Carried out at 43 sites across 19 countries	Patients were eligible if they had completed the phase 3 APOLLO or phase 2 OLE parent studies and tolerated the study drug.	Patisiran 0.3 mg/kg IV Q3W	211	55 (26%)	61.3	12 months

mBMI is defined as weight in kilograms divided by square of height in meters + albumin level in grams per liter.

hATTR, Hereditary Transthyretin Amyloidosis; LT, liver transplantation; TTR, transthyretin; IV, Intravenous; SC, Subcutaneous; Q3M, Every 3 months; Q3W, Every 3 weeks; NIS-LL, Neuropathy Impairment Score of the Lower Limbs; Norfolk QOL-DN, Norfolk Quality of Life-Diabetic Neuropathy; mBMI, Modified Body Mass Index; RNAi, RNA interference; OLE, Open-Label Extension; IU, International Units; ATTR-CM, Transthyretin Amyloid Cardiomyopathy; OLE, open-label extension.

### Main results

Coelho et al. (20) investigation focused on assessing the safety and efficacy of two RNAi therapies, ALN-TTR01 and ALN-TTR02 (Patisiran), focusing on TTR levels within individuals diagnosed with ATTRv and a control group of unaffected individuals. In the ALN-TTR01 trial involving 32 ATTRv patients, single doses of ALN-TTR01 (ranging from 0.01 to 1.0 mg/kg) or placebo were administered. Notably, the 1.0 mg/kg dose demonstrated a significant 38% mean TTR knockdown at day seven compared to the placebo, affecting both mutant and nonmutant TTR similarly. Infusion reactions of mild to moderate severity occurred in 20.8% of the ALN-TTR01 group. In the ALN-TTR02 Phase 1 trial with 17 healthy volunteers, single doses of ALN-TTR02 (ranging from 0.01 to 0.5 mg/kg) or placebo were given. The study revealed potent, dose-dependent TTR lowering, with a mean 82–87% TTR knockdown at nadir for 0.15 and 0.3 mg/kg doses. Remarkably, a durable knockdown of 56–67% persisted on day 28, with only one mild infusion reaction reported in the 0.5 mg/kg group (20).

In summary, ALN-TTR01 and ALN-TTR02 demonstrated a significant capacity to suppress TTR production via an RNAi mechanism, thereby establishing proof of concept. ALN-TTR02 exhibited notably enhanced potency compared to ALN-TTR01, and both RNAi therapies were generally well-tolerated. Additional research is crucial to assess these interventions’ clinical effectiveness and long-term safety thoroughly (20).

Coelho et al. (21) conducted research that revealed that prolonged Patisiran administration was both safe and efficacious in arresting or facilitating the advancement of polyneuropathy in individuals with ATTRv. Patisiran reduced TTR levels by approximately 82% over 24 months, resulting in an average improvement of 6.95 points in mNIS+7 compared to baseline among 19 out of 27 patients at the end of the 24 months. Throughout the duration of the study, assessments of motor function, autonomic symptoms, disease progression, and quality of life showed no notable changes. Likewise, no significant variations were observed in echocardiographic evaluations or cardiac biomarkers among the subgroup of patients with cardiac involvement. Reported adverse events were mainly mild to moderate severity, and none necessitated treatment discontinuation. Flushes and infusion reactions were the most common drug-related incidents, each accounting for 22% of the reported adverse events (21).

Suhr et al. (22) attempted to evaluate the tolerability, safety, pharmacokinetic (PK), and pharmacodynamic (PD) of Patisiran in patients with transthyretin-mediated FAP. The inclusion criteria for this study encompassed adult patients diagnosed with confirmed ATTRv. Baseline serum levels of TTR proteins exhibited uniformity across various dosage cohorts. TTR knockdown was identical between patients with the Val30Met and those with non-Val30Met genotypes. The reduction in TTR levels was strongly correlated with decreased circulating retinol-binding protein (RBP) and vitamin A. After the administration of Patisiran, the mean concentrations of the TTR small interfering RNAs (siRNAs) component decreased (22).

Nevertheless, there was no buildup of siRNAs after administering the second dose. The C_max_ and AUC0-last increased proportionally with the dose for both the first and second doses. Mild-to-moderate reaction during the infusion was the main side effect associated with their study. It is worthy of note that a few cases also experienced various adverse events, including back pain, asthenia, leukocytosis, neutrophilia, cellulitis, lymphangitis, polyuria, nausea/vomiting, facial erythema, dry mouth, pyrexia, dysphagia (22).

The study HELIOS-A was conducted by Adams et al. (13), which lasted for 18 months, and examined the safety and effectiveness of Vutrisiran for individuals with ATTRv and polyneuropathy. The investigation demonstrated that Vutrisiran exerted a favorable influence on the primary endpoint, specifically the alteration from baseline in the modified Neuropathy Impairment Score + 7 (mNIS+7) at the nine-month mark (−2.24 [Vutrisiran] and + 14.76 [placebo] (*p* = 3.54 × 10–12)). Furthermore, the compound demonstrated favorable outcomes across various secondary efficacy measures. Vutrisiran exhibited notable advancements in Norfolk QOL-DN during the ninth month compared to the external placebo cohort. The Vutrisiran group demonstrated a 53.4% enhancement, surpassing the 23.4% improvement observed in the placebo group, as indicated by the Norfolk QOL-DN score. By the eighteenth month, these figures further accentuated the positive impact of Vutrisiran, with a 56.8% improvement observed in the Vutrisiran group, in contrast to a mere 10.4% improvement in the placebo group. The 10-meter walk test (10MWT) demonstrated significant improvements at both the nine and 18-month intervals (13).

Additionally, the mNIS+7 results after 18 months showed a notable difference, with a decrease of 0.46 for Vutrisiran and an increase of 28.1 for the placebo. Notably, 48.3% of Vutrisiran recipients exhibited improvement in mNIS+7, in contrast to only 3.9% in the external placebo cohort. Furthermore, favorable results were noted in the modified Body Mass Index (mBMI) and the Rasch-built Overall Disability Scale (R-ODS) after 18 months. The majority of adverse occurrences documented exhibited mild to moderate severity and aligned with the expected course of ATTRv. Noteworthy is the absence of any occurrences necessitating cessation of drug treatment or resulting in fatalities. The research findings underscore that Vutrisiran demonstrated substantial improvements in various disease-related parameters for ATTRv compared to an external placebo while maintaining a commendable safety record (13).

Merkel et al. (23) conducted an investigation employing Indirect Treatment Comparison (ITC) methodology to evaluate the therapeutic effectiveness of Vutrisiran and Tafamidis across diverse parameters such as NIS-LL, Norfolk QOL-DN score, NIS-LL Response, and mBMI. This investigation seeks to establish the effectiveness of both Vutrisiran and Tafamidis in treating ATTRv with polyneuropathy. The empirical basis for this study is derived from the analysis of data obtained from phase three randomized controlled trials, namely HELIOS-A, APOLLO, and Fx-005 studies. These trials systematically evaluated and compared the outcomes associated with Vutrisiran and Tafamidis interventions in the context of ATTRv with polyneuropathy. The primary analysis demonstrated a statistically significant superiority of Vutrisiran compared to Tafamidis in terms of the average change from baseline at the 18-month evaluation point for two crucial outcome metrics: the NIS-LL (mean difference: −5.3, 95% CI: −9.4 to −1.2, *p* = 0.011) and the Norfolk QOL-DN (mean difference: −18.3, CI 95%: −28.6 to −8.0, *p* < 0.001). Additionally, Vutrisiran exhibited superior efficacy in the NIS-LL Responder outcome at the 18-month mark compared to Tafamidis NIS-LL response (odds ratio: 1.7). This ITC study concluded that Vutrisiran has higher efficacy in enhancing multiple measures of polyneuropathy impairment and health-related quality of life (HRQOL) compared to Tafamidis in patients with ATTRv amyloidosis with polyneuropathy (23).

Adams et al. (24) conducted a research study to examine the effectiveness of Patisiran in patients with ATTRv with polyneuropathy. All individuals involved in this research were of adult age and exhibited an NIS ranging from 5 to 130, along with a polyneuropathy disability score of 3b or below, and demonstrated sufficient liver and renal functionality. According to the research, Patisiran demonstrated significant enhancements in both the primary endpoint (mNIS+7) for 56% of the patients who received the treatment, as opposed to only 4% of those who received a placebo. Additionally, regarding the secondary endpoint, Norfolk QOL-DN score, 51% of the patients who received Patisiran displayed an improvement in their Norfolk QOL-DN score, compared to 10% of those who received a placebo. In the subset of individuals within the cardiac subpopulation, Patisiran demonstrated a superior impact on cardiac structural integrity and functionality compared to administering a placebo. It is noteworthy that both cohorts documented the occurrence of adverse events throughout the clinical trial (24).

Schmidt et al. (25) conducted a Phase 3b open-label trial to evaluate the efficacy and safety of Patisiran in patients with ATTRv amyloidosis who experienced polyneuropathy progression following liver transplantation. The study involved mature individuals who had received a liver transplant as a treatment for ATTRv amyloidosis at least a year before the study and had encountered the advancement of polyneuropathy after the transplant. The research findings revealed that Patisiran led to an average reduction of approximately 91% in TTR levels between Months 6 and 12. Additionally, at Month 12, improvements in neuropathy, quality of life, and autonomic symptoms were observed, with mean changes from baseline of −3.7 (SEM 2.7) in the NIS, −6.5 (SEM 4.9) in the Norfolk QOL-DN score, and − 5.0 (SEM 2.6) in the Composite Autonomic Symptom Score-31 (COMPASS-31). Measures of disability, assessed using the R-ODS, and nutritional status, monitored through the mBMI, remained relatively stable with mean changes from baseline of −0.1 (SEM 1.1) and + 4.4 (SEM 21.8), respectively, over the 12-month treatment period. Adverse events reported during the trial were consistent with the known safety profile of Patisiran, including diarrhea and infusion-related reactions (25).

After an 18-month retrospective study, Bleecker et al. (26) investigated 12 patients diagnosed with ATTRv who underwent Patisiran treatment. The comprehensive character of the disease is demonstrated through the manifestation of neurological and/or cardiological symptoms, as indicated by various metrics and assessments. The investigation revealed that 98.9% of the intended treatment regimens were successfully executed throughout the study (26). The median durations of treatment were 551.1 days and 700 days, respectively, spanning from 35 to 815 days. Among the patients, seven exhibited stage 1 polyneuropathy, while the remaining five presented stage 2. The polyneuropathy disability (PND) score varied from 1 to 3b, with a median NIS-LL score of 31. Eleven patients had abnormal Electromyography (EMG) results. The median Functional Independence Measurements (FIM) score was 115, and six patients had cardiac symptoms. Six patients had abnormal electrocardiogram (ECG) and left ventricular ejection fraction (LVEF) findings (26).

Fontana et al. (27) investigated for 12 months and found that Patisiran was well tolerated. The median reduction in serum TTR levels among the cohort of treated patients amounted to 86% (interquartile range [IQR]: 82 to 90%). Furthermore, it is noteworthy that 82% of the cases exhibited a TTR knockdown exceeding 80%. The administration of Patisiran therapy showed a remarkable decrease in extracellular volume (ECV), as evidenced by the adjusted mean difference between groups amounting to −6.2% (*p* = 0.001). Furthermore, there was an evident reduction in levels of N-terminal pro–B-type natriuretic peptide (NT-proBNP) linked with Patisiran treatment, as demonstrated by the adjusted mean difference between cohorts, which amounted to −1,342 ng/L (*p* = 0.012). Moreover, Patisiran therapy was significantly associated with better distances in the 6MWT, as indicated by adjusted mean differences between groups of 169 meters (*p* = 0.004). Additionally, a median reduction of 19.6% in cardiac uptake was observed, as determined through bone scintigraphy, and expressed with an IQR of 9.8 to 27.1%. In conclusion, the investigation postulated that reductions in ECV, as discerned through cardiac magnetic resonance, offered compelling evidence of regression in ATTRv affecting the heart in select patients undergoing Patisiran treatment (27). The cardiologic effects, neurologic consequences, and adverse events documented in the encompassed studies have been briefly presented in Tables 2–4, respectively.

**Table 2:** Cardiological effects.

Literature title	First author	Publication year	Cardiologic parameters
Patisiran, an RNAi Therapeutic for Hereditary Transthyretin Amyloidosis (24)	D. Adams	2018	Left ventricular wall thickness (mm): Least-squares mean difference at 18 months (Patisiran – Placebo) was −0.9 ± 0.4 mm (*p*-value: 0.02) Left ventricular longitudinal strain (%): Least-squares mean difference at 18 months (Patisiran – Placebo) was −1.37 ± 0.56% (*p*-value: 0.02) NT-proBNP^1^: Least-squares mean difference in NT-proBNP levels at 18 months (Patisiran – Placebo) was 0.45^2^ (*p*-value <0.001)
Long-term safety and efficacy of patisiran for hereditary transthyretin-mediated amyloidosis with polyneuropathy: 12-month results of an open-label extension study (28)	D. Adams	2021	Serum TTR: Within the context of the global OLE study, the administration of patisiran demonstrated a noteworthy and consistent decline in the mean concentration of serum TTR. Specifically, the APOLLO-placebo cohort exhibited a mean percentage reduction of 78.7% (SD 17.1) in TTR levels after a 6-month treatment period. This reduction was sustained in both the APOLLO-patisiran and the phase 2 OLE patisiran groups during the ongoing patisiran intervention in the global open-label extension. NYHA and NT-proBNP: Within the placebo group, alterations in NT-proBNP concentration occurred during the APOLLO study; however, the specific percentage change was not reported.
A retrospective survey of patients with hereditary transthyretin-mediated (hATTR) amyloidosis treated with patisiran in real-world clinical practice in Belgium (26)	JL. De Bleecker	2023	Last measurement vs. baseline: NYHA score (*N* = 9) Improved: 1 case (11%) Stable: 7 cases (78%) Worsened: 1 case (11%) ECG (*N* = 9) Stable: 9 cases (100%) Echocardiography^3^ (*N* = 9) Stable: 9 cases (100%)
Reduction in CMR Derived Extracellular Volume With Patisiran Indicates Cardiac Amyloid Regression (27)	*M. Fontana*	2021	Median 6MWT (m): (*p*-Value: 0.004) Group Regression Coefficient: 169 (57, 280) Median NT-proBNP (ng/l): (*p*-Value: 0.012) Group Regression Coefficient: −1,343 (−2,364 to −322) Median LVEDVi (ml/m^2): (*p*-Value: 0.047) Group Regression Coefficient: −6.165 (−12.237 to −0.094) Median ECV (%): (*p*-Value: 0.001) Group Regression Coefficient: −6.2 (−9.5 to −3.0) Other parameters had no significant difference.

1 N-terminal pro-brain natriuretic peptide (NT-proBNP) is a measure of cardiac stress that is an independent predictor of death in patients with transthyretin cardiac amyloidosis.

2 Shown is the ratio of adjusted geometric mean ratio to baseline at month 18 between the two trial groups (patisiran: placebo).

3 Echocardiography–Median/mean difference in % LVEF (min/max): 5.0/9.5 (− 5/37).

RNAi, RNA interference; mm: millimeters; NT-proBNP, N-Terminal Pro-B-Type Natriuretic Peptide; hATTR, Hereditary Transthyretin-mediated Amyloidosis; NYHA, New York Heart Association; ECG, Electrocardiography; CMR, Cardiac Magnetic Resonance; 6MWT, Six-Minute Walk Test; ng/l, nanograms per liter; LVEDVi, Left Ventricular End-Diastolic Volume Index; ml/m^2, milliliters per square meter; ECV, Extracellular Volume; OLE, Open-Label Extension; SD, Standard Deviation.

**Table 3:** Neurological effects.

Literature title	First author	Publication year	Neurological parameters
Efficacy and safety of vutrisiran for patients with hereditary transthyretin-mediated amyloidosis with polyneuropathy: a randomized clinical trial (13)	D. Adams	2022	The mean change from baseline to Month 9 (SE) for various clinical assessments: Norfolk QOL-DN: Placebo: 12.9 (2.2) Vutrisiran: −3.3 (1.7) R-ODS: Placebo: −4.9 (0.7) Vutrisiran: −0.6 (0.5) 10-MWT for gait speed (m/s): Placebo: −0.133 (0.025) Vutrisiran: −0.001 (0.019) The mean change from baseline to Month 18 (SE) for selected assessments is as follows: mNIS+7: Placebo: 28.1 (2.3) Vutrisiran: −0.46 (1.6) Norfolk QOL-DN: Placebo: 19.8 (2.6) Vutrisiran: −1.2 (1.8) R-ODS: Placebo: −9.9 (0.8) Vutrisiran: −1.5 (0.6) 10-MWT for gait speed (m/s): Placebo: −0.264 (0.036) Vutrisiran: −0.024 (0.025)
Indirect treatment comparison (ITC) of the efficacy of vutrisiran and Tafamidis for hereditary transthyretin-mediated amyloidosis with polyneuropathy (23)	M. Merkel	2023	Mean differences from baseline to Month 18 are presented as follows: NIS-LL: Vutrisiran vs. Placebo: −8.3 Vutrisiran vs. Tafamidis: −5.3 Vutrisiran vs. Placebo: −10.7 Norfolk QOL-DN: Vutrisiran vs. Placebo: −23.5 Vutrisiran vs. Tafamidis: −18.3 Vutrisiran vs. Placebo: −21.9 Odds Ratio NIS-LL Responder^1^ at Month 18: Vutrisiran vs. Placebo: 3.4 Vutrisiran vs. Tafamidis: 1.7 Vutrisiran vs. Placebo: 3.7
Patisiran, an RNAi Therapeutic for Hereditary Transthyretin Amyloidosis (24)	D. Adams	2018	mNIS+7^2^: Least-squares mean Difference at 18 months (Patisiran – Placebo): −34.0 *P* Value: <0.001
A phase II, open-label, extension study of long-term patisiran treatment in patients with hereditary transthyretin-mediated (hATTR) amyloidosis (21)	T. Coelho	2020	Mean (SEM) changes in mNIS+7 components from baseline to Month 24: Total: −6.95 (2.03) NIS: +1.92 (1.84) NIS + 7: +2.54 (1.84)
Long-term safety and efficacy of patisiran for hereditary transthyretin-mediated amyloidosis with polyneuropathy: 12-month results of an open-label extension study (28)	D.Adams	2021	Norfolk QOL-DN: Sustained enhancements in Norfolk QOL-DN were evident within the APOLLO-patisiran group during the global Open-Label Extension (OLE) at the 12-month mark, relative to the baseline established in the APOLLO trial (mean change of −3.9, 95% CI −8.1 to 0.3). Notably, patients in the APOLLO-placebo group exhibited improved Norfolk QOL-DN during the global OLE, as reflected by a mean change from global OLE enrollment to 12 months of −4.5 (95% CI −9.6 to 0.7), effectively arresting the deterioration witnessed during the APOLLO trial. PND: The amelioration in polyneuropathy, as indicated by a negative change in the mean modified Neuropathy Impairment Score + 7 (mNIS+7) relative to both the APOLLO and phase 2 OLE baselines, was sustained at the global OLE 12-month juncture. Specifically, the mean change for APOLLO-patisiran was −4.0 (95% CI −7.7 to −0.3), and for phase 2 OLE patisiran, it was −4.7 (95% CI −11.9 to 2.4). However, the mean mNIS+7 score did not revert to the APOLLO baseline, with a mean change from APOLLO baseline to global OLE 12 months of +24.0 (95% CI 15.4 to 32.5). COMPASS-31: The patisiran group demonstrated improvement in autonomic function. The mean change in autonomic function from global OLE enrollment to 12 months was −3.7 (95% CI −8.0 to 0.6).
A retrospective survey of patients with hereditary transthyretin-mediated (hATTR) amyloidosis treated with patisiran in real-world clinical practice in Belgium (26)	JL. De Bleecker	2023	Comparison of Last Measurement to Baseline in Percentage (%): FAP: Participants (*N* = 9) Stable: 8 cases (89%) Worsened: 1 case (11%) PND: Participants (*N* = 4) Improved: 1 case (11%) Stable: 3 cases (33%) NIS-LL: Participants (*N* = 4) Improved: 2 cases (22%) Stable: 2 cases (22%) EMG: Participants (*N* = 9) Improved or Stable: 9 cases (99%) FIM Score: Participants (*N* = 9) Improved: 2 cases (22%) Stable: 6 cases (67%) Worsened: 1 case (11%) Orthostatic Hypotension: Participants (*N* = 9) Improved: 2 cases (22%) Stable: 7 cases (78%)

1 e NIS-LL Responder was defined as mean change in NIS-LL of < 2 points increase or any level of decrease from baseline in NIS-LL at month 18.

2 modified Neuropathy Impairment Score+7 is a composite measure of neuropathy that assesses motor, sensory, and autonomic neuropathy (range, 0 to 304, with higher scores indicating more impairment); FIM score, Functional Independence Measure score.

**Table 4:** Adverse events.

Literature title	First author	Publication year	Adverse events
Efficacy and safety of vutrisiran for patients with hereditary transthyretin-mediated amyloidosis with polyneuropathy: a randomized clinical trial (13)	D. Adams	2022	Any AE: Placebo: 75 cases (97.4%) Vutrisiran: 119 cases (97.5%) Patisiran: 41 cases (97.6%) Death: Placebo: 6 cases (7.8%) Vutrisiran: 2 cases (1.6%) Patisiran: 3 cases (7.1%) Any serious AE: Placebo: 31 cases (40.3%) Vutrisiran: 32 cases (26.2%) Patisiran: 18 cases (42.9%) Any severe AE: Placebo: 28 cases (36.4%) Vutrisiran: 19 cases (15.6%) Patisiran: 16 cases (38.1%) AEs leading to treatment discontinuation: Placebo: 11 cases (14.3%) Vutrisiran: 3 cases (2.5%) Patisiran: 3 cases (7.1%) AEs leading to stopping study participation: Placebo: 9 cases (11.7%) Vutrisiran: 3 cases (2.5%) - Patisiran: 2 cases (4.8%)
Patisiran, an RNAi Therapeutic, for Hereditary Transthyretin Amyloidosis (24)	D. Adams	2018	Any AE: Placebo: 75 cases (97%) Patisiran: 143 cases (97) Death: Placebo: 6 cases (8%) Patisiran: 7 cases (5%) Any Serious AE: Placebo: 31 cases (40%) Patisiran: 54 cases (36%) Any Severe AE: Placebo: 28 cases (36%) Patisiran: 42 cases (28%)
A phase II, open-label, extension study of long-term patisiran treatment in patients with hereditary transthyretin-mediated (hATTR) amyloidosis (21)	T. Coelho	2020	Any AE: 26 cases (96%) Any AE related to the study drug: 16 cases (59%) Any serious AE: 7 cases (26%) Death: 2 cases (7%) Any AE leading to discontinuation: 2 cases (7%)
Efficacy and safety of patisiran for familial amyloidotic polyneuropathy: a phase II multi-dose study (22)	O. Suhr	2015	The most common side effect related to the study was a mild-to-moderate infusion-related reaction. Some other adverse events, including back pain, asthenia, leukocytosis, neutrophilia, cellulitis, lymphangitis, polyuria, nausea/vomiting, facial erythema, dry mouth, pyrexia, and dysphagia, were observed in a few patients.
Safety and Efficacy of RNAi Therapy for Transthyretin Amyloidosis (20)	T. Coelho	2013	Mild-to-moderate infusion-related reactions: ALN-TTR01 at doses of at least 0.4 mg: 20.8% ALN-TTR02 at a dose of 0.5 mg per kilogram: the single participant (7.7%) No drug-related serious adverse events or any study-drug discontinuations because of adverse events No significant changes in hematologic, liver, or renal measurements or thyroid function No drug-related serious adverse events were reported in either group

QOL-DN, Quality of Life in Diabetic Neuropathy; R-ODS, Rasch-built Overall Disability Scale; 10-MWT, 10-Meter Walk Test; mNIS + 7, modified Neuropathy Impairment Score + 7; ITC, Indirect Treatment Comparison; NIS-LL, Neuropathy Impairment Score of the Lower Limbs; CI, Confidence Interval; OLE, Open-Label Extension; PND, Polyneuropathy Disability; APOLLO, A Study of Patisiran in Patients with Hereditary ATTR Amyloidosis; FAP, Familial Amyloid Polyneuropathy; EMG, Electromyography; FIM, Functional Independence Measure, AE, Adverse Event; hATTR, Hereditary Transthyretin; RNAi, RNA Interference.

Adams et al. (28) extended the phase 3 open-label clinical trial over 12 months. Data were collected systematically at regular intervals, revealing persistent enhancements in polyneuropathy measures following treatment with Patisiran. The analysis also revealed improved QOL, autonomic function, disability, and nutritional condition. Nevertheless, despite a consistent enhancement in polyneuropathy, it did not revert to the baseline levels observed in prior studies. The average alteration in autonomic function demonstrated a decrease of −3.7 following 12 months of treatment. Furthermore, the levels of TTR exhibited a significant reduction of 78% after 6 months of treatment. The study’s primary objective was to evaluate the safety and tolerability of prolonged Patisiran administration. Secondary objectives included investigating disease progression, assessing quality of life, and evaluating nutritional well-being (28).

### Risk of bias

These investigations offer noteworthy discoveries; nonetheless, it is essential to recognize the conceivable hazards linked to conflicts of interest and biases that could have influenced the research outcomes. An identifiable source of bias in these inquiries is the sponsorship received from specific pharmaceutical companies, introducing the potential for partiality in study design, execution, and reporting. Furthermore, researchers may be inclined to disclose positive results to ensure continued financial support from these entities. To alleviate the risk of bias and conflicts of interest, researchers ought to openly divulge potential conflicts and institute measures to ensure the objectivity and impartiality of the research. The sponsor of the trials is indeed a pharmaceutical company, but this is inevitable since a pharmaceutical company initiates most phase I-III trials.

In the study by Schmidt et al. (25), the authors disclosed conflicts of interest, including employment and stock ownership with Alnylam Pharmaceuticals, as well as consulting fees, honoraria, and research funding from Alnylam Pharmaceuticals, Pfizer Inc., Akcea Therapeutics, Ionis Pharmaceuticals, and Eidos. Additionally, Alnylam Pharmaceuticals supported related research by Adams et al. (13, 24, 28), Merckle et al. (23), and Coelho et al. (20, 21). Furthermore, Adams et al. (28) also reported financial support from Pfizer, while Coelho et al. (20) disclosed funding from the National Institute for Health Research (NIHR) Biomedical Research Center at Guy’s and St. Thomas’ NHS Foundation Trust and King’s College London, specifically allocated to Dr. Mant (20).

The authors of the investigation by Suhr et al. (22) revealed financial affiliations with pharmaceutical enterprises among the involved parties. O Suhr is actively engaged in clinical investigations in collaboration with Pfizer and Alnylam Pharmaceuticals. T Coelho’s medical institution has received remunerations from multiple corporate entities, and Coelho personally has received financial backing from Alnylam Pharmaceuticals and Pfizer. J Buades, J Pouget, and I Conceicao are affiliated with Alnylam Pharmaceuticals, whereas J Berk and M Waddington-Cruz have received financial support from pharmaceutical corporations.

In a study by Bleeker et al. (26), various institutions such as Sanofi-Genzyme, CSL Behring, Pfizer, Alnylam, Biogen, Roche, and Alexion provided compensation between 2008 and 2021. On the other hand, the study by Fontana et al. (27) was financially supported by the British Heart Foundation, with reference number FS/18/21/33447. Furthermore, the authors reported consulting affiliations with Alnylam, Akcea, Eidos, Intellia, Pfizer, and Alexion.

## Discussion

ATTRv causes multisystem dysfunction due to peripheral and autonomic neuropathy and/or cardiomyopathy. This significantly and progressively impacts physical functioning and QOL (24, 29, 30). Vutrisiran and Patisiran represent RNAi treatments aimed at diminishing the levels of pathogenic TTR protein by suppressing the expression of the TTR gene in individuals afflicted with ATTRv (13, 24).

This systematic review recommends using Vutrisiran and Patisiran in ATTRv. The recommendation is based on outcomes of the latest studies that showed notable enhancements across various disease-relevant parameters in individuals with ATTRv (13, 20–24, 26–28). The results indicate that Patisiran (20, 21, 24, 25, 28) and Vutrisiran (13) considerably enhanced the mNIS+7 and Norfolk QOL-DN results. Vutrisiran (13) also demonstrated significant enhancements in the 10MWT, mBMI, and R-ODS. At the same time, Patisiran showed substantial improvements in the NIS-LL Field (25, 26), autonomic function, disability, and nutritional situation (20, 21, 25, 28), as well as reduced neurofilament light (NfL) levels (31). Both pharmaceutical interventions were well-tolerated among the study participants, and most adverse incidents exhibited mild to moderate severity (13, 20–24, 28).

Merkel et al. (23) conducted an analysis using phase three randomized controlled trials. They used a Bucher analysis to compare the effects of Vutrisiran and Tafamidis’ treatment. The results indicated that Vutrisiran is more effective than Tafamidis in treating symptoms associated with polyneuropathy manifestations, HRQOL, nutritional status, NIS-LL, Norfolk QOL-DN, and mBMI in patients with a diagnosis of ATTRv amyloidosis. They observed disparity in efficacy holds clinical significance, suggesting Vutrisiran’s potential as a preferable option for maintaining physical function and enhancing the QOL in this patient population (23).

Schmidt et al. (25) demonstrated that 12 months of Patisiran treatment led to a 91.0% median reduction in serum TTR levels. Significant improvements were observed in neuropathy (NIS), quality of life (Norfolk QOL-DN), and autonomic symptoms (COMPASS-31). Additionally, disability (R-ODS) and nutritional status (mBMI) remained stable throughout the study period. Patisiran demonstrated a favorable safety profile with no new safety concerns emerging. These findings underscore the efficacy of Patisiran in the management of polyneuropathy in patients with ATTRv following liver transplantation.

Furthermore, the second interim analysis of the Global OLE study (31) provides empirical evidence regarding the long-term administration of Patisiran treatment spanning 48 months. The results demonstrate sustained enhancements in mNIS+7 and Norfolk QOL-DN scores over an additional 24 months of Patisiran treatment, accompanied by a safety profile deemed acceptable and consistent with previous reports spanning up to 12 months (28). Additionally, analytical studies showed that, In the APOLLO and HELIOS-A trials, NfL levels significantly decreased at 4 and 18 months in patients treated with Patisiran or Vutrisiran (31, 32).

The APOLLO and phase 2 OLE investigations have demonstrated that Patisiran therapy significantly diminishes NfL concentrations and enhances neuropathy outcomes as evaluated by mNIS+7 and QOL measures in contrast to initial assessments (21). The findings remained consistent throughout an additional 24-month treatment period in the Global OLE investigation, demonstrating a correlation between the impact of Patisiran on NfL levels and its clinical efficacy (24). After a nine-month treatment regimen, Patisiran demonstrated notable superiority over a placebo in enhancing the QOL. By the 18-month, enhancements were evident across all QOL metrics, particularly within the predetermined cardiac subgroup, as delineated in the APOLLO study. These findings underscore the advantageous impact of Patisiran on QOL among individuals afflicted with both polyneuropathy and cardiomyopathy, conditions known to diminish overall well-being substantially (33). Postural blood pressure (PBP) evaluations investigate the impact of RNAi medications such as Patisiran and Vutrisiran on autonomic functioning among individuals diagnosed with ATTRv accompanied by polyneuropathy. The observed elevation of PBP levels into symptomatic thresholds among untreated individuals underscores the importance of prompt intervention measures (34).

Patisiran and Vutrisiran exert an influence on cardiac symptoms and findings as well. Notably, Vutrisiran therapy has demonstrated a beneficial effect on NT-ProBNP levels and echocardiographic parameters within the subset of patients with cardiac involvement. It also led to a reduction in cardiac uptake of 99mTc, suggesting a decrease in cardiac amyloid. Nevertheless, the clinical significance remains uncertain at present (35). Patisiran treatment has reduced NT-proBNP (24, 27, 28), TTR levels (28), cardiac uptake (27), and abnormal ECG (26). Additionally, it demonstrated enhanced cardiac morphology and performance, evidenced by notable variances in the average thickness of the left ventricular wall and longitudinal strain (26) and an increase in 6MWT distances (27). Cardiac magnetic resonance (CMR) is a superior alternative for assessing myocardial tissue and estimating cardiac amyloid burden (27).

Depending on the phenotype and country regulations, Tafamidis, Diflunisal, Patisiran, Inotersen, Vutrisiran, and Eplontersen can be used for ATTRv amyloidosis. Patisiran is administered at 0.3 mg/kg via intravenous infusion lasting 80 min every 3 weeks (24). In contrast, Vutrisiran is a novel therapeutic agent that operates through a similar mechanism and is administered at a dosage of 25 mg via subcutaneous injection every 3 months (Q3M) (13).

Regarding cost, Vutrisiran is estimated to be more cost-effective than Patisiran due to its less frequent dosing schedule and lower administration costs. The cost savings for Vutrisiran depend on factors such as how long it takes to administer Patisiran and which healthcare professional distributes it. Considering these factors, a cost comparison suggests that Vutrisiran is more cost-effective than Patisiran and is therefore recommended (36).

This systematic review article acknowledges several limitations that impede a comprehensive evaluation of Patisiran and Vutrisiran’s effects on amyloidosis polyneuropathy and myocardiopathies. Firstly, the scarcity of studies available to the researchers limits the scope of the analysis. Secondly, heterogeneity in participant numbers across studies challenges comparing results. Thirdly, most studies were funded by Anylham, a drug manufacturer, which raises the possibility of publication bias. Finally, incomplete follow-up periods in a series of studies further complicate the interpretation of findings. Given these limitations, it is recommended that large-scale studies be conducted to provide a more robust understanding of the effects of Vutrisiran and Patrisiran on these conditions.

## Conclusion

This comprehensive analysis evaluated the effectiveness and safety of RNA interference treatments, Patisiran and Vutrisiran, in managing ATTRv amyloidosis. The study consistently demonstrated favorable results, revealing that Patisiran and Vutrisiran significantly reduce pathogenic TTR protein levels. This reduction significantly improved neuropathy parameters, quality of life, and cardiac symptoms among patients with ATTRv amyloidosis. The findings strongly support using these RNA interference therapies for controlling the multisystem dysfunction associated with the disease. However, it is imperative to approach the interpretation of the results with caution due to potential biases stemming from funding sources and variations in study designs. Extended investigations with prolonged observation periods and diverse cohorts are essential to enhance our comprehensive understanding of these interventions’ enduring effectiveness and safety.

## Data Availability

The original contributions presented in the study are included in the article/Supplementary material, further inquiries can be directed to the corresponding author.

## Footnotes

^1^ https://osf.io/evwtd

^2^ https://jbi.global/critical-appraisal-tools
