# Supplementary material for: Assessing the effectiveness and safety of Patisiran and Vutrisiran in ATTRv amyloidosis with polyneuropathy: a systematic review
Source: Front Neurol. 2024 Sep 2;15:1465747. doi: 10.3389/fneur.2024.1465747 (PMC11402727; doi:10.3389/fneur.2024.1465747)
Supplement: Supplementary file 1 [file Data_Sheet_1.docx]

Records identified from:

PubMed/Medline (n = 72)

Embase (n = 352)

Web of Science (n = 171)

Cochrane Central (n = 17)

Google Scholar (n = 246)

Records removed *before screening*:

Duplicate records removed (n = 182)

Records screened

(n = 676)

Records excluded**

(n = 577)

Reports assessed for eligibility

(n = 99)

Reports excluded: (n = 89)

Reasons:

Irrelevant outcome

Studies included in review

(n = 10)

**Identification of studies via databases and registers**

**Identification**

**Screening**

**Included**

**Fig.1** The PRISMA diagram for the search and study selection process

**Table 1: The Search strategy in databases conducted on September 16th, 2023**

| **Search Engine** | **Search Strategy** | **Number of results** |
| --- | --- | --- |
| PubMed | #1:("patisiran" [Supplementary Concept]) AND ("Amyloid Neuropathies, Familial"[Mesh])  #2:("Amyloid Neuropathies, Familial"[Mesh]) AND ((vutrisiran[Title/Abstract]) OR (Amvuttra[Title/Abstract]) OR (ALN-TTRSC02[Title/Abstract]))  Final:#1 OR #2 | 72 |
| Scopus | ( ( TITLE-ABS-KEY ( neuropath* ) ) OR ( TITLE-ABS-KEY ( polyneur* ) ) ) AND ( ( TITLE-ABS-KEY ( patisiran ) ) OR ( TITLE-ABS-KEY ( onpattro ) ) OR ( TITLE-ABS-KEY ( aln-ttr02 ) ) OR ( TITLE-ABS-KEY ( vutrisiran ) ) OR ( TITLE-ABS-KEY ( amvuttra ) ) OR ( TITLE-ABS-KEY ( aln-ttrsc02 ) ) OR ( TITLE-ABS-KEY ( aln-ttrsc02 ) ) ) | 352 |
| Web of Science | #1: patisiran (Topic) OR Onpattro (Topic) OR ALN-TTR02 (Topic) OR vutrisiran (Topic) OR Amvuttra (Topic) OR ALN-TTRSC02 (Topic)  #2: polyneur* (Topic) OR neuropath* (Topic)  Final:#1 AND #2 | 171 |
| Cochrane (central) | #1: MeSH descriptor: [Amyloid Neuropathies] explode all trees  #2: (patisiran):ti,ab,kw OR (Onpattro):ti,ab,kw OR (ALN-TTR02):ti,ab,kw OR (vutrisiran):ti,ab,kw OR (Amvuttra):ti,ab,kw OR ALN-TTRSC02  Final:#1 AND #2 | 17 |
| Google Scholar | allintitle: patisiran OR Onpattro OR "ALN TTR02" OR vutrisiran OR Amvuttra OR "ALN TTRSC02" | 246 |

**JBI Critical Appraisal Checklist for cohort studies**

Reviewer: Fatemeh Esmaeilpour moallem Date: 2023/18/12

Author: Bleecker et al Year: 2023 Record Number_________

|  | Yes | No | Unclear | Not applicable |
| --- | --- | --- | --- | --- |
| 1. Were the two groups similar and recruited from the same population? | ◼ | □ | □ | □ |
| 1. Were the exposures measured similarly to assign people to both exposed and unexposed groups? | ◼ | □ | □ | □ |
| 1. Was the exposure measured in a valid and reliable way? | ◼ | □ | □ | □ |
| 1. Were confounding factors identified? | ◼ | □ | □ | □ |
| 1. Were strategies to deal with confounding factors stated? | ◼ | □ | □ | □ |
| 1. Were the groups/participants free of the outcome at the start of the study (or at the moment of exposure)? | □ | □ | ◼ | □ |
| 1. Were the outcomes measured in a valid and reliable way? | ◼ | □ | □ | □ |
| 1. Was the follow up time reported and sufficient to be long enough for outcomes to occur? | □ | ◼ | □ | □ |
| 1. Was follow up complete, and if not, were the reasons to loss to follow up described and explored? | ◼ | □ | □ | □ |
| 1. Were strategies to address incomplete follow up utilized? | ◼ | □ | □ | □ |
| 1. Was appropriate statistical analysis used? | ◼ | □ | □ | □ |

Overall appraisal: Include ◼ Exclude □ Seek further info □

Comments (Including reason for exclusion)

_______11/9__________________________________________________________________________________

**JBI Critical Appraisal Checklist for cohort studies**

Reviewer: Fatemeh Esmaeilpour moallem Date: 2023/18/12

Author: Fontana et al Year: 2021 Record Number

|  | Yes | No | Unclear | Not applicable |
| --- | --- | --- | --- | --- |
| 1. Were the two groups similar and recruited from the same population? | ◼ | □ | □ | □ |
| 1. Were the exposures measured similarly to assign people to both exposed and unexposed groups? | ◼ | □ | □ | □ |
| 1. Was the exposure measured in a valid and reliable way? | ◼ | □ | □ | □ |
| 1. Were confounding factors identified? | □ | ◼ | □ | □ |
| 1. Were strategies to deal with confounding factors stated? | □ | ◼ | □ | □ |
| 1. Were the groups/participants free of the outcome at the start of the study (or at the moment of exposure)? | ◼ | □ | □ | □ |
| 1. Were the outcomes measured in a valid and reliable way? | ◼ | □ | □ | □ |
| 1. Was the follow up time reported and sufficient to be long enough for outcomes to occur? | ◼ | □ | □ | □ |
| 1. Was follow up complete, and if not, were the reasons to loss to follow up described and explored? | ◼ | □ | □ | □ |
| 1. Were strategies to address incomplete follow up utilized? | ◼ | □ | □ | □ |
| 1. Was appropriate statistical analysis used? | ◼ | □ | □ | □ |

Overall appraisal: Include ◼ Exclude □ Seek further info □

Comments (Including reason for exclusion)

____9/11______________________________________________________________________________________

**JBI Critical Appraisal Checklist for cohort studies**

Reviewer ____Mohamma Sadra Gholami Chahkand_________

Date______2023/24/12___________

Author__ Coelho et al. ________________ Year___2020___ Record Number_________

|  | Yes | No | Unclear | Not applicable |
| --- | --- | --- | --- | --- |
| 1. Were the two groups similar and recruited from the same population? | ◼ | □ | □ | □ |
| 1. Were the exposures measured similarly to assign people to both exposed and unexposed groups? | ◼ | □ | □ | □ |
| 1. Was the exposure measured in a valid and reliable way? | ◼ | □ | □ | □ |
| 1. Were confounding factors identified? | ◼ | □ | □ | □ |
| 1. Were strategies to deal with confounding factors stated? | ◼ | □ | □ | □ |
| 1. Were the groups/participants free of the outcome at the start of the study (or at the moment of exposure)? | □ | □ | ◼ | □ |
| 1. Were the outcomes measured in a valid and reliable way? | ◼ | □ | □ | □ |
| 1. Was the follow up time reported and sufficient to be long enough for outcomes to occur? | ◼ | □ | □ | □ |
| 1. Was follow up complete, and if not, were the reasons to loss to follow up described and explored? | ◼ | □ | □ | □ |
| 1. Were strategies to address incomplete follow up utilized? | ◼ | □ | □ | □ |
| 1. Was appropriate statistical analysis used? | ◼ | □ | □ | □ |

Overall appraisal: Include ◼ Exclude □ Seek further info □

Comments (Including reason for exclusion)

___11/10____________________________________________________________________________________

**JBI Critical Appraisal Checklist for
analytical cross-sectional studies**

Reviewer: ____Mohammad Sadra Gholami Chahkand_________ Date______2023/29/12___________

Author__ Merkel et al. ________________ Year___2023___ Record Number_________

|  | Yes | No | Unclear | Not applicable |
| --- | --- | --- | --- | --- |
| 1. Were the criteria for inclusion in the sample clearly defined? | ◼ | □ | □ | □ |
| 1. Were the study subjects and the setting described in detail? | ◼ | □ | □ | □ |
| 1. Was the exposure measured in a valid and reliable way? | ◼ | □ | □ | □ |
| 1. Were objective, standard criteria used for measurement of the condition? | ◼ | □ | □ | □ |
| 1. Were confounding factors identified? | ◼ | □ | □ | □ |
| 1. Were strategies to deal with confounding factors stated? | ◼ | □ | □ | □ |
| 1. Were the outcomes measured in a valid and reliable way? | ◼ | □ | □ | □ |
| 1. Was appropriate statistical analysis used? | ◼ | □ | □ | □ |

Overall appraisal: Include ◼ Exclude □ Seek further info □

Comments (Including reason for exclusion)

__________8/8_________________________________________________________________________________

JBI CRITICAL APPRAISAL TOOL FOR assessment of risk of bias for randomized controlled trials

| **Assessor:** Mohammad Amin Karimi | | | | | | **Date of Appraisal:** 11 February 2022 | | | **Record Number:** | | | | | |
| --- | --- | --- | --- | --- | --- | --- | --- | --- | --- | --- | --- | --- | --- | --- |
| **Study Author:** Schmidt et al. | | | | | | **Study Title:** Patisiran treatment in patients with hereditary transthyretin mediated amyloidosis with polyneuropathy after liver transplantation | | | **Study Year:** 2022 | | | | | |
|  | | | | | |  | | |  | | | | | |
| **Internal Validity** | | | | | | | | **Choice - Comments/Justification** | | **Yes** | **No** | **Unclear** | **N/A** | |
| **Bias related to selection and allocation** | | | | | | | | | | | | | | |
| **1** | **Was true randomization used for assignment of participants to treatment groups?** | | | | | | |  | |  |  |  |  | |
| **2** | **Was allocation to treatment groups concealed?** | | | | | | |  | |  |  |  |  | |
| **3** | **Were treatment groups similar at the baseline?** | | | | | | |  | |  |  |  |  | |
| **Bias related to administration of intervention/exposure** | | | | | | | | | | | | | | |
| **4** | **Were participants blind to treatment assignment?** | | | | | | |  | |  |  |  |  | |
| **5** | **Were those delivering the treatment blind to treatment assignment?** | | | | | | |  | |  |  |  |  | |
| **6** | **Were treatment groups treated identically other than the intervention of interest?** | | | | | | |  | |  |  |  |  | |
| **Bias related to assessment, detection and measurement of the outcome** | | | | | | | | | | | | | | |
| **7** | **Were outcome assessors blind to treatment assignment?** | | | | | | |  | | **Yes** | **No** | **Unclear** | **N/A** | |
|  | **Outcomes** | | | | | | |  | |  |  |  |  | |
|  |  | | | | | | |  | |  |  |  |  | |
| **8** | **Were outcomes measured in the same way for treatment groups?** | | | | | | |  | | **Yes** | **No** | **Unclear** | **N/A** | |
|  | **Outcomes** | | | | | | |  | |  |  |  |  | |
|  |  | | | | | | |  | |  |  |  |  | |
| **9** | **Were outcomes measured in a reliable way** | | | | | | |  | | **Yes** | **No** | **Unclear** | **N/A** | |
|  | **Outcomes** | | | | | | |  | |  |  |  |  | |
|  |  | | | | | | |  | |  | | | | |
| **Bias related to participant retention** | | | | | | | | | | | | | | |
| **10** | **Was follow up complete and if not, were differences between groups in terms of their follow up adequately described and analysed?** | | | | | | |  | |  | | | | |
|  | **Outcomes** | | | | | | |  | | **Yes** | **No** | **Unclear** | **N/A** | |
|  |  | Results | | | | | |  | |  |  |  |  | |
|  |  |  | | | | | | | |  |  |  |  | |
|  | **Statistical Conclusion Validity** | | | | | | | | |  |  |  |  | |
| **11** | **Were participants analysed in the groups to which they were randomized?** | | | | | | |  | |  | | | | |
|  | **Outcomes** | | |  | | | |  | | **Yes** | **No** | **Unclear** | **N/A** | |
|  |  | Results | | | | | |  | |  |  |  |  | |
|  |  | | | | | | | | |  |  |  | |  |
| **12** | **Was appropriate statistical analysis used?** | | | | | | |  | |  |  |  | |  |
|  | **Outcomes** | | | | | | |  | | **Yes** | **No** | **Unclear** | | **N/A** |
|  |  | Results | | | | | |  | |  |  |  | |  |
|  |  | | | | | | |  | |  |  |  | |  |
|  |  | | | | | | |  | | **Yes** | **No** | **Unclear** | | **N/A** |
| **13** | **Was the trial design appropriate and any deviations from the standard RCT design (individual randomization, parallel groups) accounted for in the conduct and analysis of the trial?** | | | | | | |  | |  |  |  | |  |
| **Overall appraisal:** | | | **Include:** | | **Exclude:** | | **Seek Further Info:** | | | | | | | |
| **Comments:** | | | | | | | | | | | | | | |
| JBI CRITICAL APPRAISAL TOOL FOR assessment of risk of bias for randomized controlled trials | | | | | | | | | | | | | | |

| **Assessor:** Mohammad Javad Emami Kazemabad | | | | | | **Date of Appraisal:** 3 Jan 2024 | | | **Record Number:** | | | | | |
| --- | --- | --- | --- | --- | --- | --- | --- | --- | --- | --- | --- | --- | --- | --- |
| **Study Author:** Suhr et al. | | | | | | **Study Title:** Efficacy and safety of patisiran for familial amyloidotic polyneuropathy: a phase II multi-dose study | | | **Study Year:** 2015 | | | | | |
|  | | | | | |  | | |  | | | | | |
| **Internal Validity** | | | | | | | | **Choice - Comments/Justification** | | **Yes** | **No** | **Unclear** | **N/A** | |
| **Bias related to selection and allocation** | | | | | | | | | | | | | | |
| **1** | **Was true randomization used for assignment of participants to treatment groups?** | | | | | | |  | |  |  |  |  | |
| **2** | **Was allocation to treatment groups concealed?** | | | | | | |  | |  |  |  |  | |
| **3** | **Were treatment groups similar at the baseline?** | | | | | | |  | |  |  |  |  | |
| **Bias related to administration of intervention/exposure** | | | | | | | | | | | | | | |
| **4** | **Were participants blind to treatment assignment?** | | | | | | |  | |  |  |  |  | |
| **5** | **Were those delivering the treatment blind to treatment assignment?** | | | | | | |  | |  |  |  |  | |
| **6** | **Were treatment groups treated identically other than the intervention of interest?** | | | | | | |  | |  |  |  |  | |
| **Bias related to assessment, detection and measurement of the outcome** | | | | | | | | | | | | | | |
| **7** | **Were outcome assessors blind to treatment assignment?** | | | | | | |  | | **Yes** | **No** | **Unclear** | **N/A** | |
|  | **Outcomes** | | | | | | |  | |  |  |  |  | |
|  |  | | | | | | |  | |  |  |  |  | |
| **8** | **Were outcomes measured in the same way for treatment groups?** | | | | | | |  | | **Yes** | **No** | **Unclear** | **N/A** | |
|  | **Outcomes** | | | | | | |  | |  |  |  |  | |
|  |  | | | | | | |  | |  |  |  |  | |
| **9** | **Were outcomes measured in a reliable way** | | | | | | |  | | **Yes** | **No** | **Unclear** | **N/A** | |
|  | **Outcomes** | | | | | | |  | |  |  |  |  | |
|  |  | | | | | | |  | |  | | | | |
| **Bias related to participant retention** | | | | | | | | | | | | | | |
| **10** | **Was follow up complete and if not, were differences between groups in terms of their follow up adequately described and analysed?** | | | | | | |  | |  | | | | |
|  | **Outcomes** | | | | | | |  | | **Yes** | **No** | **Unclear** | **N/A** | |
|  |  | Results | | | | | |  | |  |  |  |  | |
|  | **Statistical Conclusion Validity** | | | | | | | | |  |  |  |  | |
| **11** | **Were participants analysed in the groups to which they were randomized?** | | | | | | |  | |  | | | | |
|  | **Outcomes** | | |  | | | |  | | **Yes** | **No** | **Unclear** | **N/A** | |
|  |  | Results | | | | | |  | |  |  |  |  | |
|  |  | | | | | | | | |  |  |  | |  |
| **12** | **Was appropriate statistical analysis used?** | | | | | | |  | |  |  |  | |  |
|  | **Outcomes** | | | | | | |  | | **Yes** | **No** | **Unclear** | | **N/A** |
|  |  | Results | | | | | |  | |  |  |  | |  |
|  |  | | | | | | |  | |  |  |  | |  |
|  |  | | | | | | |  | | **Yes** | **No** | **Unclear** | | **N/A** |
| **13** | **Was the trial design appropriate and any deviations from the standard RCT design (individual randomization, parallel groups) accounted for in the conduct and analysis of the trial?** | | | | | | |  | |  |  |  | |  |
| **Overall appraisal:** | | | **Include:** | | **Exclude:** | | **Seek Further Info:** | | | | | | | |
| **Comments:** | | | | | | | | | | | | | | |
|  | | | | | | | | | | | | | | |

JBI CRITICAL APPRAISAL TOOL FOR assessment of risk of bias for randomized controlled trials

| **Assessor:** Mohammad Amin Karimi | | | | | | **Date of Appraisal:** 4 Jan 2024 | | | **Record Number:** | | | | | |
| --- | --- | --- | --- | --- | --- | --- | --- | --- | --- | --- | --- | --- | --- | --- |
| **Study Author:** Adams et al. | | | | | | **Study Title**: Long-term safety and efficacy of patisiran for hereditary transthyretin-mediated amyloidosis with polyneuropathy: 12-month results of an open-label extension study | | | **Study Year:** 2021 | | | | | |
|  | | | | | |  | | |  | | | | | |
| **Internal Validity** | | | | | | | | **Choice - Comments/Justification** | | **Yes** | **No** | **Unclear** | **N/A** | |
| **Bias related to selection and allocation** | | | | | | | | | | | | | | |
| **1** | **Was true randomization used for assignment of participants to treatment groups?** | | | | | | |  | |  |  |  |  | |
| **2** | **Was allocation to treatment groups concealed?** | | | | | | |  | |  |  |  |  | |
| **3** | **Were treatment groups similar at the baseline?** | | | | | | |  | |  |  |  |  | |
| **Bias related to administration of intervention/exposure** | | | | | | | | | | | | | | |
| **4** | **Were participants blind to treatment assignment?** | | | | | | |  | |  |  |  |  | |
| **5** | **Were those delivering the treatment blind to treatment assignment?** | | | | | | |  | |  |  |  |  | |
| **6** | **Were treatment groups treated identically other than the intervention of interest?** | | | | | | |  | |  |  |  |  | |
| **Bias related to assessment, detection and measurement of the outcome** | | | | | | | | | | | | | | |
| **7** | **Were outcome assessors blind to treatment assignment?** | | | | | | |  | | **Yes** | **No** | **Unclear** | **N/A** | |
|  | **Outcomes** | | | | | | |  | |  |  |  |  | |
|  |  | | | | | | |  | |  |  |  |  | |
| **8** | **Were outcomes measured in the same way for treatment groups?** | | | | | | |  | | **Yes** | **No** | **Unclear** | **N/A** | |
|  | **Outcomes** | | | | | | |  | |  |  |  |  | |
|  |  | | | | | | |  | |  |  |  |  | |
| **9** | **Were outcomes measured in a reliable way** | | | | | | |  | | **Yes** | **No** | **Unclear** | **N/A** | |
|  | **Outcomes** | | | | | | |  | |  |  |  |  | |
|  |  | | | | | | |  | |  | | | | |
| **Bias related to participant retention** | | | | | | | | | | | | | | |
| **10** | **Was follow up complete and if not, were differences between groups in terms of their follow up adequately described and analysed?** | | | | | | |  | |  | | | | |
|  | **Outcomes** | | | | | | |  | | **Yes** | **No** | **Unclear** | **N/A** | |
|  |  | Results | | | | | |  | |  |  |  |  | |
|  | **Statistical Conclusion Validity** | | | | | | | | |  |  |  |  | |
| **11** | **Were participants analysed in the groups to which they were randomized?** | | | | | | |  | |  | | | | |
|  | **Outcomes** | | |  | | | |  | | **Yes** | **No** | **Unclear** | **N/A** | |
|  |  | Results | | | | | |  | |  |  |  |  | |
|  |  | | | | | | | | |  |  |  | |  |
| **12** | **Was appropriate statistical analysis used?** | | | | | | |  | |  |  |  | |  |
|  | **Outcomes** | | | | | | |  | | **Yes** | **No** | **Unclear** | | **N/A** |
|  |  | Results | | | | | |  | |  |  |  | |  |
|  |  | | | | | | |  | |  |  |  | |  |
|  |  | | | | | | |  | | **Yes** | **No** | **Unclear** | | **N/A** |
| **13** | **Was the trial design appropriate and any deviations from the standard RCT design (individual randomization, parallel groups) accounted for in the conduct and analysis of the trial?** | | | | | | |  | |  |  |  | |  |
| **Overall appraisal:** | | | **Include:** | | **Exclude:** | | **Seek Further Info:** | | | | | | | |
| **Comments:** | | | | | | | | | | | | | | |
|  | | | | | | | | | | | | | | |

JBI CRITICAL APPRAISAL TOOL FOR assessment of risk of bias for randomized controlled trials

| **Assessor:** Mohammad Amin Karimi | | | | | | **Date of Appraisal:** 3 Jan 2024 | | | **Record Number:** | | | | | |
| --- | --- | --- | --- | --- | --- | --- | --- | --- | --- | --- | --- | --- | --- | --- |
| **Study Author:** Coelho et al. | | | | | | **Study Title:** Safety and Efficacy of RNAi Therapy for Transthyretin Amyloidosis | | | **Study Year:** 2013 | | | | | |
|  | | | | | |  | | |  | | | | | |
| **Internal Validity** | | | | | | | | **Choice - Comments/Justification** | | **Yes** | **No** | **Unclear** | **N/A** | |
| **Bias related to selection and allocation** | | | | | | | | | | | | | | |
| **1** | **Was true randomization used for assignment of participants to treatment groups?** | | | | | | |  | |  |  |  |  | |
| **2** | **Was allocation to treatment groups concealed?** | | | | | | |  | |  |  |  |  | |
| **3** | **Were treatment groups similar at the baseline?** | | | | | | |  | |  |  |  |  | |
| **Bias related to administration of intervention/exposure** | | | | | | | | | | | | | | |
| **4** | **Were participants blind to treatment assignment?** | | | | | | |  | |  |  |  |  | |
| **5** | **Were those delivering the treatment blind to treatment assignment?** | | | | | | |  | |  |  |  |  | |
| **6** | **Were treatment groups treated identically other than the intervention of interest?** | | | | | | |  | |  |  |  |  | |
| **Bias related to assessment, detection and measurement of the outcome** | | | | | | | | | | | | | | |
| **7** | **Were outcome assessors blind to treatment assignment?** | | | | | | |  | | **Yes** | **No** | **Unclear** | **N/A** | |
|  | **Outcomes** | | | | | | |  | |  |  |  |  | |
|  |  | | | | | | |  | |  |  |  |  | |
| **8** | **Were outcomes measured in the same way for treatment groups?** | | | | | | |  | | **Yes** | **No** | **Unclear** | **N/A** | |
|  | **Outcomes** | | | | | | |  | |  |  |  |  | |
|  |  | | | | | | |  | |  |  |  |  | |
| **9** | **Were outcomes measured in a reliable way** | | | | | | |  | | **Yes** | **No** | **Unclear** | **N/A** | |
|  | **Outcomes** | | | | | | |  | |  |  |  |  | |
|  |  | | | | | | |  | |  | | | | |
| **Bias related to participant retention** | | | | | | | | | | | | | | |
| **10** | **Was follow up complete and if not, were differences between groups in terms of their follow up adequately described and analysed?** | | | | | | |  | |  | | | | |
|  | **Outcomes** | | | | | | |  | | **Yes** | **No** | **Unclear** | **N/A** | |
|  |  | Results | | | | | |  | |  |  |  |  | |
|  | **Statistical Conclusion Validity** | | | | | | | | |  |  |  |  | |
| **11** | **Were participants analysed in the groups to which they were randomized?** | | | | | | |  | |  | | | | |
|  | **Outcomes** | | |  | | | |  | | **Yes** | **No** | **Unclear** | **N/A** | |
|  |  | Results | | | | | |  | |  |  |  |  | |
|  |  | | | | | | | | |  |  |  | |  |
| **12** | **Was appropriate statistical analysis used?** | | | | | | |  | |  |  |  | |  |
|  | **Outcomes** | | | | | | |  | | **Yes** | **No** | **Unclear** | | **N/A** |
|  |  | Results | | | | | |  | |  |  |  | |  |
|  |  | | | | | | |  | |  |  |  | |  |
|  |  | | | | | | |  | | **Yes** | **No** | **Unclear** | | **N/A** |
| **13** | **Was the trial design appropriate and any deviations from the standard RCT design (individual randomization, parallel groups) accounted for in the conduct and analysis of the trial?** | | | | | | |  | |  |  |  | |  |
| **Overall appraisal:** | | | **Include:** | | **Exclude:** | | **Seek Further Info:** | | | | | | | |
| **Comments:** | | | | | | | | | | | | | | |
|  | | | | | | | | | | | | | | |

JBI CRITICAL APPRAISAL TOOL FOR assessment of risk of bias for randomized controlled trials

| **Assessor:** Mohammad Amin Karimi | | | | | | **Date of Appraisal:** 4 Jan 2024 | | | **Record Number:** | | | | | |
| --- | --- | --- | --- | --- | --- | --- | --- | --- | --- | --- | --- | --- | --- | --- |
| **Study Author:** Adams et al. | | | | | | **Study Title**: Efficacy and safety of vutrisiran for patients with hereditary transthyretin-mediated amyloidosis with polyneuropathy: a randomized clinical trial | | | **Study Year:** 2023 | | | | | |
|  | | | | | |  | | |  | | | | | |
| **Internal Validity** | | | | | | | | **Choice - Comments/Justification** | | **Yes** | **No** | **Unclear** | **N/A** | |
| **Bias related to selection and allocation** | | | | | | | | | | | | | | |
| **1** | **Was true randomization used for assignment of participants to treatment groups?** | | | | | | |  | |  |  |  |  | |
| **2** | **Was allocation to treatment groups concealed?** | | | | | | |  | |  |  |  |  | |
| **3** | **Were treatment groups similar at the baseline?** | | | | | | |  | |  |  |  |  | |
| **Bias related to administration of intervention/exposure** | | | | | | | | | | | | | | |
| **4** | **Were participants blind to treatment assignment?** | | | | | | |  | |  |  |  |  | |
| **5** | **Were those delivering the treatment blind to treatment assignment?** | | | | | | |  | |  |  |  |  | |
| **6** | **Were treatment groups treated identically other than the intervention of interest?** | | | | | | |  | |  |  |  |  | |
| **Bias related to assessment, detection and measurement of the outcome** | | | | | | | | | | | | | | |
| **7** | **Were outcome assessors blind to treatment assignment?** | | | | | | |  | | **Yes** | **No** | **Unclear** | **N/A** | |
|  | **Outcomes** | | | | | | |  | |  |  |  |  | |
|  |  | | | | | | |  | |  |  |  |  | |
| **8** | **Were outcomes measured in the same way for treatment groups?** | | | | | | |  | | **Yes** | **No** | **Unclear** | **N/A** | |
|  | **Outcomes** | | | | | | |  | |  |  |  |  | |
|  |  | | | | | | |  | |  |  |  |  | |
| **9** | **Were outcomes measured in a reliable way** | | | | | | |  | | **Yes** | **No** | **Unclear** | **N/A** | |
|  | **Outcomes** | | | | | | |  | |  |  |  |  | |
|  |  | | | | | | |  | |  | | | | |
| **Bias related to participant retention** | | | | | | | | | | | | | | |
| **10** | **Was follow up complete and if not, were differences between groups in terms of their follow up adequately described and analysed?** | | | | | | |  | |  | | | | |
|  | **Outcomes** | | | | | | |  | | **Yes** | **No** | **Unclear** | **N/A** | |
|  |  | Results | | | | | |  | |  |  |  |  | |
|  | **Statistical Conclusion Validity** | | | | | | | | |  |  |  |  | |
| **11** | **Were participants analysed in the groups to which they were randomized?** | | | | | | |  | |  | | | | |
|  | **Outcomes** | | |  | | | |  | | **Yes** | **No** | **Unclear** | **N/A** | |
|  |  | Results | | | | | |  | |  |  |  |  | |
|  |  | | | | | | | | |  |  |  | |  |
| **12** | **Was appropriate statistical analysis used?** | | | | | | |  | |  |  |  | |  |
|  | **Outcomes** | | | | | | |  | | **Yes** | **No** | **Unclear** | | **N/A** |
|  |  | Results | | | | | |  | |  |  |  | |  |
|  |  | | | | | | |  | |  |  |  | |  |
|  |  | | | | | | |  | | **Yes** | **No** | **Unclear** | | **N/A** |
| **13** | **Was the trial design appropriate and any deviations from the standard RCT design (individual randomization, parallel groups) accounted for in the conduct and analysis of the trial?** | | | | | | |  | |  |  |  | |  |
| **Overall appraisal:** | | | **Include:** | | **Exclude:** | | **Seek Further Info:** | | | | | | | |
| **Comments:** | | | | | | | | | | | | | | |

JBI CRITICAL APPRAISAL TOOL FOR assessment of risk of bias for randomized controlled trials

| **Assessor:** Mohammad Amin Karimi | | | | | | **Date of Appraisal:** 4 Jan 2024 | | | **Record Number:** | | | | | |
| --- | --- | --- | --- | --- | --- | --- | --- | --- | --- | --- | --- | --- | --- | --- |
| **Study Author:** Adams et al. | | | | | | **Study Title**: Patisiran, an RNAi Therapeutic, for Hereditary Transthyretin Amyloidosis | | | **Study Year:** 2018 | | | | | |
|  | | | | | |  | | |  | | | | | |
| **Internal Validity** | | | | | | | | **Choice - Comments/Justification** | | **Yes** | **No** | **Unclear** | **N/A** | |
| **Bias related to selection and allocation** | | | | | | | | | | | | | | |
| **1** | **Was true randomization used for assignment of participants to treatment groups?** | | | | | | |  | |  |  |  |  | |
| **2** | **Was allocation to treatment groups concealed?** | | | | | | |  | |  |  |  |  | |
| **3** | **Were treatment groups similar at the baseline?** | | | | | | |  | |  |  |  |  | |
| **Bias related to administration of intervention/exposure** | | | | | | | | | | | | | | |
| **4** | **Were participants blind to treatment assignment?** | | | | | | |  | |  |  |  |  | |
| **5** | **Were those delivering the treatment blind to treatment assignment?** | | | | | | |  | |  |  |  |  | |
| **6** | **Were treatment groups treated identically other than the intervention of interest?** | | | | | | |  | |  |  |  |  | |
| **Bias related to assessment, detection and measurement of the outcome** | | | | | | | | | | | | | | |
| **7** | **Were outcome assessors blind to treatment assignment?** | | | | | | |  | | **Yes** | **No** | **Unclear** | **N/A** | |
|  | **Outcomes** | | | | | | |  | |  |  |  |  | |
|  |  | | | | | | |  | |  |  |  |  | |
| **8** | **Were outcomes measured in the same way for treatment groups?** | | | | | | |  | | **Yes** | **No** | **Unclear** | **N/A** | |
|  | **Outcomes** | | | | | | |  | |  |  |  |  | |
|  |  | | | | | | |  | |  |  |  |  | |
| **9** | **Were outcomes measured in a reliable way** | | | | | | |  | | **Yes** | **No** | **Unclear** | **N/A** | |
|  | **Outcomes** | | | | | | |  | |  |  |  |  | |
|  |  | | | | | | |  | |  | | | | |
| **Bias related to participant retention** | | | | | | | | | | | | | | |
| **10** | **Was follow up complete and if not, were differences between groups in terms of their follow up adequately described and analysed?** | | | | | | |  | |  | | | | |
|  | **Outcomes** | | | | | | |  | | **Yes** | **No** | **Unclear** | **N/A** | |
|  |  | Results | | | | | |  | |  |  |  |  | |
|  | **Statistical Conclusion Validity** | | | | | | | | |  |  |  |  | |
| **11** | **Were participants analysed in the groups to which they were randomized?** | | | | | | |  | |  | | | | |
|  | **Outcomes** | | |  | | | |  | | **Yes** | **No** | **Unclear** | **N/A** | |
|  |  | Results | | | | | |  | |  |  |  |  | |
|  |  | | | | | | | | |  |  |  | |  |
| **12** | **Was appropriate statistical analysis used?** | | | | | | |  | |  |  |  | |  |
|  | **Outcomes** | | | | | | |  | | **Yes** | **No** | **Unclear** | | **N/A** |
|  |  | Results | | | | | |  | |  |  |  | |  |
|  |  | | | | | | |  | |  |  |  | |  |
|  |  | | | | | | |  | | **Yes** | **No** | **Unclear** | | **N/A** |
| **13** | **Was the trial design appropriate and any deviations from the standard RCT design (individual randomization, parallel groups) accounted for in the conduct and analysis of the trial?** | | | | | | |  | |  |  |  | |  |
| **Overall appraisal:** | | | **Include:** | | **Exclude:** | | **Seek Further Info:** | | | | | | | |
| **Comments:** | | | | | | | | | | | | | | |
